# AQP3 and AQP5—Potential Regulators of Redox Status in Breast Cancer

**DOI:** 10.3390/molecules26092613

**Published:** 2021-04-29

**Authors:** Lidija Milković, Ana Čipak Gašparović

**Affiliations:** Division of Molecular Medicine, Ruđer Bošković Institute, HR-10000 Zagreb, Croatia; lidija.milkovic@irb.hr

**Keywords:** AQP3, AQP5, oxidative stress

## Abstract

Breast cancer is still one of the leading causes of mortality in the female population. Despite the campaigns for early detection, the improvement in procedures and treatment, drastic improvement in survival rate is omitted. Discovery of aquaporins, at first described as cellular plumbing system, opened new insights in processes which contribute to cancer cell motility and proliferation. As we discover new pathways activated by aquaporins, the more we realize the complexity of biological processes and the necessity to fully understand the pathways affected by specific aquaporin in order to gain the desired outcome–remission of the disease. Among the 13 human aquaporins, AQP3 and AQP5 were shown to be significantly upregulated in breast cancer indicating their role in the development of this malignancy. Therefore, these two aquaporins will be discussed for their involvement in breast cancer development, regulation of oxidative stress and redox signalling pathways leading to possibly targeting them for new therapies.

## 1. Introduction

Despite the progress in research and treatment procedures, cancer still remains the leading cause of death. Today, cancer is targeted via different approaches which is determined by diagnosis, tumour marker expression, and specific mutations. One of the first approaches was targeting highly proliferating (cancer) cells and this is the basic mechanism of conventional chemotherapy. Conventional chemotherapy, such as anthracyclines, taxols, cisplatin or other platinum-containing drugs, targets either DNA replication or repair, or interferes with tubulins, disabling proper chromatid exchange in mitosis. Oxidative stress commonly accompanies these therapies, which damage cancer cells, but also normal cells, thereby causing side effects such as cardiotoxicity or ototoxicity [1,2,3].

Oxidative stress is both a cause and consequence of pathological states. On the other hand, numerous physiological processes depend on mild oxidative stress and certainly redox signalling is now recognized for its role in physiology [4]. Although ROS were at first thought to be by-products of metabolic pathways, now we are repurposing proteins that produce and control ROS movements thereby contributing to and controlling redox signalling [5]. Aquaporins are one of these molecules which were attributed as water channels making a breakthrough in our knowledge of water movement or transport across the cellular membrane [6]. In the light of accumulating evidence on the role of aquaporin 3 and aquaporin 5 in breast cancer, we will bring an overview of current knowledge of these two aquaporins and their role in the regulation of redox signalling in breast cancer.

## 2. Aquaporins

Water movement in and out of the cells is one of the most important biological processes since it is the base for cell movement, regulation of cytoplasmic viscosity, and consequently kinetics of signal transduction. Water was supposed to enter through the cell membrane via diffusion, but this process has slow kinetics, and the changes of volume that occur due to water movement are quite rapid indicating a certain level of active regulation. The discovery of aquaporins, the first one described was CHIP28/AQP1 [7], opened new research perspective. At first, aquaporins were figuratively described as cell’s plumbing system focusing on their role in water transport [8]. Today, we are distinguishing this family according to molecules channelled to aquaporins and aquaglyceroporins. Aquaporin number varies between the species, e.g., *Saccharomyces cerevisiae* has two orthodox (Aqy1 and Aqy2) and two glyceroaquaporins (YFL054Cp and Fps1), *Escherichia coli* has one orthodox, AqpZ, and one structurally similar to glyceroaquaporins, GlpF, while plants can have more than 35 isoforms [9]. 

Today we know 13 human aquaporins (AQP0-QP12), each one with a unique structure and cellular and subcellular location and role [10]. Aquaporins are grouped according to their permeability and primary structure as follows: orthodox aquaporins (AQP0, AQP1, AQP2, AQP4, AQP5, AQP6, and AQP8) which channel primarily water, glyceroaquaporins (AQP3, AQP7, AQP9, and AQP10) which channel primarily glycerol, and unorthodox or S-aquaporins (AQP11 and AQP12) [11,12]. The unorthodox S-aquaporins or super-aquaporins are located strictly inside the cell, on the membranes of organelles [13]. This group is found in animal cells but not in plants, bacteria no fungi, and has low homology with the other family members [14]. Interestingly, unorthodox aquaporins were initially attributed to channel water, but AQP11 was shown also to facilitate glycerol transport [14]. Still, their major role is the regulation of organelle volume and intra-vesicular transport [12,15]. As stated, the initial grouping of aquaporins defined the water and glycerol channels, but both of these groups also channel other small molecules (ions and other polar molecules, as well as gases) [16,17]. In the light of new targets for transport through aquaporins, hydrogen peroxide emerged due to its relevance in biological processes and signalling. Therefore, Henzler and Steudle proposed a new term “peroxiporins” which refers to aquaporins that facilitate hydrogen peroxide flux [18,19]. Peroxiporins are members of all three previously mentioned groups and are as follows AQP0, AQP1, AQP3, AQP5, AQP8, AQP9, and AQP11 [19,20].

Aquaporins are highly conserved transmembrane channels built as tetramers, with each monomer consisting of about 320 amino acids, and of 28kDa molecular weight [10]. Each monomer has six transmembrane domains connected with five loops (A-E) [6]. Another motif, asparagine-proline-alanine (NPA) is present on loop B on the cytoplasmic side and loop E on extracellular side is common to both, orthodox and aquaglyceroporins. NPA motif serves as structure maintenance motif of each monomer, while loop D regulates gating of the pore [21]. In addition to structure maintenance, loop B and loop E, each form pseudo transmembrane segment [22]. Regulation sequences of aquaporins are located in loops, specifically loop E, which contains sites for inhibitors, Hg+ and tetraethylammonium, and loop D, which is sensitive to protonation and thereby regulates gating of the pore [21]. In contrast to ion channels where the channel is centrally situated, each monomer of aquaporin is a channel of its own, regulated independently of other monomers. The tetramer centre is a channel itself, mostly hydrophobic, and channels gasses, such as CO_2_, nitric oxide, and ions; which of these species would be channelled depends specifically on each aquaporin [23,24,25,26,27,28].

**Figure 1:**
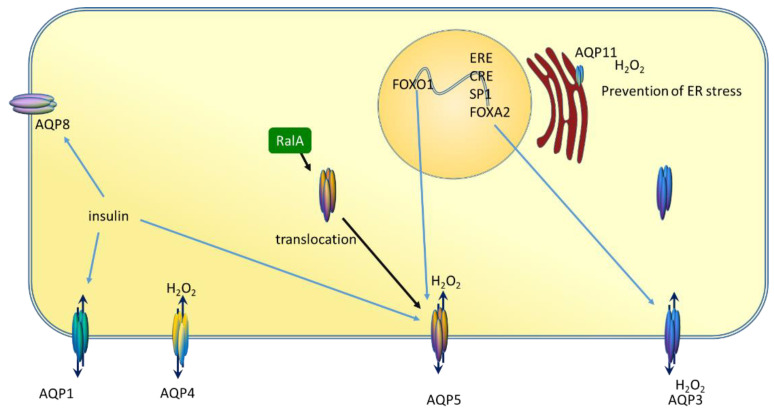
Regulation of different aquaporins-peroxiporins. Aquaporins are regulated by several transcription factors as well as hormones. Insulin increases the expression of AQP1, AQP5, and AQP8. AQP3 has several response elements in its promoter region (oestrogen response element, ERE, cAMP response element, CRE), as well as transcription binding sites (SP1, FOXA2). An additional level of regulation is translocation to the cell membrane, as observed for AQP5. AQP11 is a member of the S-aquaporin family which channels H_2_O_2_ and is located on the endoplasmic reticulum (ER). AQP11 channels H_2_O_2_ thereby preventing ER stress.

Transcriptional regulation of aquaporins (Figure 1) is still not completely investigated, but AQP3 has several response elements and transcription factor binding sites in the promoter region, such as oestrogen response element (ERE), ROR/REV-ERB-response element (RORE), SP1 site, FOXA1 site [29]. In the salivary gland, FOXO1 was shown to be a direct regulator of AQP5 expression [30]. Additionally, insulin can upregulate the expression of AQP1, AQP5, and AQP8 in the submandibular glands of diabetic rats [31]. These data indicate that aquaporins can be regulated by different stimuli further exerting protective effects on the targeted cell.

Aquaporins are regulated post-translationally by protein trafficking from the intracellular deposits and transport to the plasma membrane when needed [32]. Trafficking may depend on the phosphorylation of monomers, AQP2 needs phosphorylation of at least three monomers to determine its position in the plasma membrane [33]. In prostate cancer cells, PC-3, AQP3 is translocated from the cytoplasm to the cell membrane after silencing of RAS like proto-oncogene A (RalA) [34]. Regulation of the flux through the channel is referred to as gating and is regulated by pH, phosphorylation, temperature, membrane tension, solvent gradient, and pressure [24,35,36]. Orthodox aquaporins are known for the quickest response to stimuli by changing the permeability which implicates sophisticated regulation. Regarding the link between structure and function, each monomer functions independently of others, therefore it is not clear why they form tetramers, especially as there is no evidence of cooperative interdependence in the quaternal structure [37]. Even more, aquaporins are considered to be homotetramer, but heterotetramers were found for AQP1 and AQP4 [38,39]. 

Aquaporins control water movement across the membrane, but also they control the osmotic pressure through the regulation of intracellular glycerol concentration [40]. Control of water movement and osmotic pressure is further linked to their involvement in cellular functions, such as migration, proliferation, and adhesion [41]. During migration, aquaporins selectively transport water due to osmotic gradient which is achieved by actin depolarisation. Water enters the cell at the leading site causing local membrane expansion which is then compensated by actin rearrangement for the maintenance of the membrane integrity [42]. The mechanism by which aquaporins contribute to cell proliferation is not simple and unambiguous for each aquaporin. Namely, the inhibition of AQP1 inhibits proliferation and migration of HT29 cells, which have a high basal expression of AQP1, while this inhibition does not affect HCT116 cells, with low expression of AQP1 [43]. In addition to AQP1, AQP3 is also related to cell proliferation giving correlation of AQP3 overexpression and increased proliferation of gastric cancer cells SGC7901 and MGC803, while its downregulation had the opposite effect [44]. Taken that in gastric cancer APQ3 is significantly higher than in normal gastric mucosa and was correlated to EMT proteins in gastric cancer tissues, overexpression of AQP3 is linked to poorer prognosis of these patients. Therefore, in gastric cancer, upregulation of these two aquaporins is linked to a more malignant phenotype, which is achieved by activation of ERK and Ras, as well as PI3K/AKT/Snail signalling pathway [44,45]. In lung cancer stem cells AQP3 silencing caused upregulation of Wnt/glycogen synthase kinase-3β (GSK-3β)/β-catenin pathway implying its role in reducing the activity of this signalling pathway and, thereby, inhibiting apoptosis and reducing differentiation of lung cancer cells keeping stemness of these cell [46]. AQP3 can, therefore, through inhibition of apoptosis and reduction in differentiation support stemness of these lung cancer cells, which implies the use of APQ3 for determining the malignant potential and recurrence of the primary disease [46]. These studies indicate the complexity of aquaporins in their cellular functions and involvement in signalling pathways.

## 3. Oxidative Stress and Antioxidative Defence

One of the important factors in the development of different pathologies is oxidative stress, an imbalance in cellular redox homeostasis [47]. This imbalance can occur due to decreased antioxidant defence or due to increased production of reactive oxygen species (ROS). At first, oxidative stress was considered to be a stressful and damaging condition [48,49]. Today, we know that this point is not so straight forward, and oxidative stress is considered as altered balance which can have either positive or negative consequences [50]. These two options are denoted as eustress and distress, where distress is damaging stress with possibly lethal consequences, and eustress is hormesis, adaptive biological response to stress condition [51,52]. Eustress (hormesis) came in the focus of the research by opening numerous questions on mechanisms by which this adaptation is achieved. There are several review papers that nicely provide an overview of this topic and a historical timeline of all the major points and discoveries leading to our current perception of oxidative stress [53,54,55,56].

The concept of eustress refers to ROS as a factors that contributes to physiological processes in the cell or organism and moving our understanding of their role away from exclusively damaging factors. Mild oxidative stress, a term that is referring to low levels of ROS, is very useful because it stimulates the defence mechanisms [57]. An example of this is the exercise: during exercise the ROS production increases which stimulates the cellular antioxidative system [57]. However, if vitamin C is consumed before the activity, it will neutralize ROS produced and they will fail to stimulate the cellular antioxidative system, diminishing the positive effect of the exercise [58,59]. Increasing oxidative stress and entering the “moderate” oxidative stress activates a whole different set of proteins, stimulating inflammation and changing the expression of cytokines and chemokines [60]. Of course, an additional increase in oxidative stress inevitably leads to cell death [61]. Therefore, oscillations of oxidative stress are very welcomed by means of adaptation to environmental stress building cellular antioxidant defence system and are called the “Goldilocks Zone” referring to the story of Goldilocks and her search for “just right” conditions [55].

As stress conditions disturb this balance and shift the cell to the so called “red zone”, it is expected that the oxidative stress levels are related to the development of different pathologies, but making them an important mechanism in treating pathologies as well. Still, when treating pathologies, especially cancer, adaptation to oxidative stress is a big obstacle as cancer cells adapt and become resistant to therapy making it inefficient.

ROS and other electrophiles are highly reactive and cause oxidation of all cellular macromolecules, DNA, proteins, and lipids [62]. ROS introduce single- and double- strand breaks in DNA, and oxidize the bases which results in mutations. If not lethal, these DNA damages cause inactivation or overexpression of genes that regulate the cell cycle and lead to tumour development. Oxidation of protein disrupts their function. Lipids are vulnerable to peroxidation causing cell death due to disturbance of the physical barrier of the cell. Unlike DNA mutations and protein oxidation, lipid peroxidation is an autocatalytic process which is multiplying and expands the damage [47]. Especially vulnerable to lipid peroxidation are polyunsaturated fatty acids (PUFA) due to the double bond which is highly reactive with ROS. Lipid peroxidation is a self-multiplying process and has to be stopped by antioxidants, but it also results in the production of highly reactive aldehydes [47]. These aldehydes are stable enough to diffuse from the sight of the origin and further react with proteins, thereby modifying their reactivity and function finally affecting cellular processes [47,63,64].

As mentioned above, positive effects of mild stress are achieved by adaptation and include the nuclear factor erythroid 2 (NF-E2)-related factor 2-Kelch-like ECH-associated protein 1 (NRF2-KEAP1) pathway [52]. NRF2 is a transcription factor which is, in non-stimulating conditions, located in the cytoplasm bound to KEAP1 [65]. In the complex with KEAP1, NRF2 is ubiquitinated by Cul3-ubiquitin E2 ligase and is degraded in the proteasome [66]. If ROS or electrophiles are present in the cell, they oxidize the disulphide bond between two cysteines in KEAP1. The newly formed disulphide bond changes KEAP1 conformation which releases NRF2. NRF2 now translocates to the nucleus and binds to small MAF protein and induces transcription of antioxidant genes. NRF2 target genes are involved in glutathione synthesis (glutamate-cysteine ligase, both, catalytic and modifier subunits), in ROS detoxification (thioredoxin reductase 1, peroxiredoxin 1), xenobiotics detoxification (NQO1, NAD(P)H quinone dehydrogenase 1, glutathione-S-transferase), but also in drug transport (multidrug resistance-associated proteins, MRP) [65]. In addition, another protein family, Forkhead box O (FOXO) proteins, also contribute to antioxidative protection [67]. Among the antioxidant genes activated by the FOXO family are catalase, manganese-dependent superoxide dismutase (MnSOD), DNA damage binding protein 1 (DDB1), Fas ligand (FasL), cyclin-dependent kinase B1 (KIP1, p27), and protein for drug export, ABC1 [68].

Both, NRF2 and FOXO proteins are regulated by the PI3K/AKT signalling pathway [67,69]. Interestingly, NRF2 is activated directly by PI3K [69], but can be also activated indirectly, by 4-hydroxynonenal (HNE) activation of atypical protein kinase C (PKC) [70] and ERK [71]. FOXO family is negatively regulated by PI3K pathway, while JNK serves as a positive regulator by phosphorylating them upon the stress signal and thereby activating them [67]. Unlike NRF2, FOXO activation depends on the severity of stress, if stress levels are low, antioxidative defence mechanisms are activated, and if levels are high, apoptosis is activated [72].

## 4. Aquaporins, Oxidative Stress, and Cancer

As already described, one of the possible mechanisms by which aquaporins achieve their role in proliferation, differentiation, and apoptosis, is the regulation of small molecule transport such as hydrogen peroxide (H_2_O_2_), nitric oxide (NO), urea, and CO_2_ [23,24,25]. As hydrogen peroxide and nitric oxide regulate and modulate redox signalling pathways thereby regulating proliferation, differentiation, and apoptosis, regulation of these molecules intake can directly or indirectly contribute to the modulation of these processes resulting in tumour growth and development.

As mentioned, tumour development and progression can be a result of oxidative stress. Therefore, factors that regulate oxidative stress highly influence tumour development as well as its fate. Interestingly enough, these protective factors in many cases are actually double edge sword. This situation especially refers to NRF2, which builds up the antioxidative defence of the cell. In normal cells this is a positive set of reactions that protects from malignant transformation, while in tumour cells NRF2 leads to protection from the therapy [65,73]. Additionally, the NRF2 pathway affects and promotes tumorigenesis not only directly by activation in tumour cells, but also indirectly through cancer associated fibroblasts (CAF). Tumour cells interact with normal cells in their microenvironment, changing normal fibroblasts to CAF which are reprogrammed to support tumour growth. The CAF reprogramming occurs by activation of p62, which then targets KEAP1 to lysosomal degradation. Degradation of KEAP1 activates NRF2 and enhances transcription of ATF6 finally mediating ER stress response [74]. Evidence also suggests that FOXO, a tumour suppressor, can activate tumour resistance mechanisms, but this can happen only in combination with other events in the cell [67].

In the light of antioxidative defence acting as a double edged sword, aquaporins introduce a new moment by regulating the flux of H_2_O_2_. Delicate regulation of intracellular levels of H_2_O_2_ opens possibilities in the stimulation of proliferation and survival mechanism for tumour cells, leading to resistance and increased mobility. Aquaporins were found to be upregulated in numerous tumours [28,75,76] opening possibilities to target them as a part of tumour therapy. The exact mechanisms and signalling pathways affected by aquaporins are still to be determined, but it is not a simple and straightforward interaction. The increased presence of particular aquaporin in the cell membrane controls H_2_O_2_ flux, as well as water flux, and other small molecules mentioned above. Import and export of these small molecules, in addition to water and H_2_O_2_, can also modify signalling pathways involved in proliferation, differentiation and migration. Additionally, glycerol intake could also be one of the factors by which aquaporins achieve stimulation of proliferation intake [75]. Therefore, we will further discuss two aquaporins AQP3 and AQP5, representing glyceroaquaporin and orthodox aquaporin in details for their role in breast cancer.

## 5. AQP3 and Breast Cancer

AQP3 is a member of aquaglyceroporin, and, as such, facilitates glycerol transport [77] in addition to water [78]. Although some authors state that AQP3 is a weak water channel [46], in the kidney, AQP3 is constitutively active together with AQP4 [78] thereby regulating water excretion. Beside the role in physiology, its role in cancer was recognized very early for skin cancer [79]. Soon, evidence of AQP3 overexpression in several other types of cancers, including breast cancer, accumulated [80,81,82]. 

**Figure 2:**
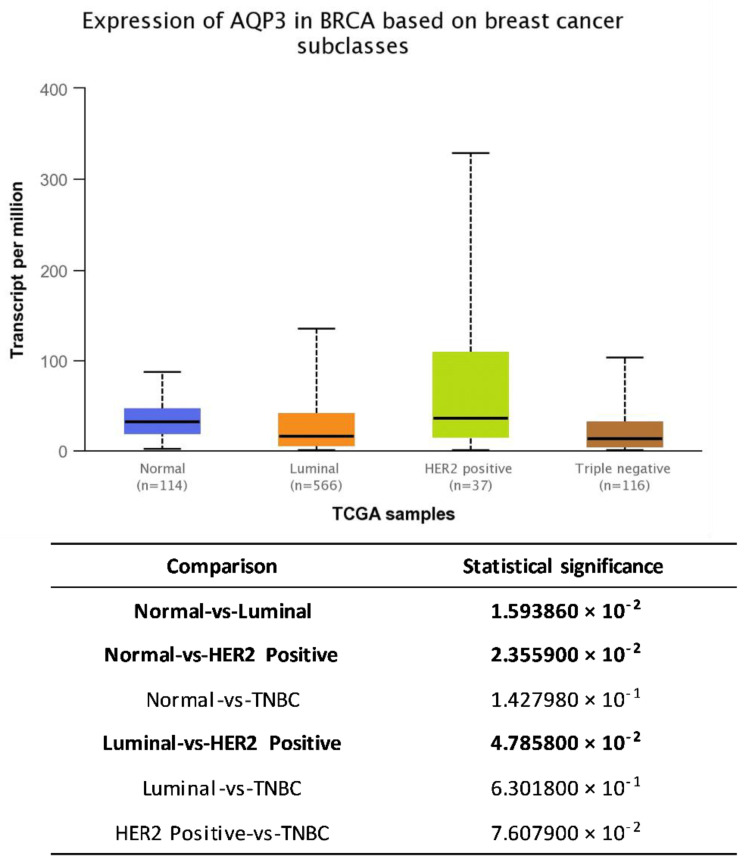
The expression of AQP3 in normal and breast cancer tissue subdivided according to expression of hormone and HER2 receptors retrieved from UALCAN [83].

Analysis of genomics data from The Cancer Genome Atlas (TCGA) project freely available from web-portal UALCAN [83] revealed pattern of AQP3 in normal (median 31.64 transcripts per million (TPM) (1.448–87.454 TPM)) vs. different subclasses of breast cancer (luminal–16.183 TPM (0.363–134.985 TPM), HER2-positive-36.481 TPM (0.521–329.342 TPM), TNBC-13.484 TPM (0.713–103.167 TPM) (Figure 2).

**Figure 3:**
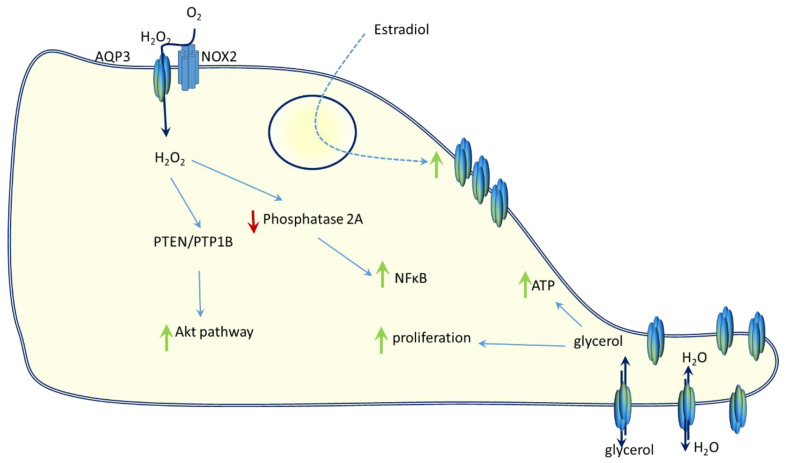
AQP3 involvement in cellular signalling pathways and processes. AQP3 respond to estradiol stimuli and increases expression. IT is also located in the membrane by the NOX2 and imports H_2_O_2_. H_2_O_2_ then oxidizes PTEN and activates AKT signalling pathways. AQP3 also channels extracellular H_2_O_2_ which inhibits phosphatase A and thereby activates NFκB. Increased positioning of AQP3 on the leading side regulates water and glycerol intake which leads to lamellipodia formation and cellular migration. Additionally, glycerol intake by AQP3 increases intracellular ATP and causes proliferation.

Once recognized as overexpressed in cancer, AQP3 was getting in the focus as a possible prognostic marker for triple negative breast cancer together with AQP5 [84], as well as for HER2 positive early breast cancer [85]. In parallel, it was shown that the *AQP3* gene has an oestrogen-response element and responds to oestrogen stimuli by increasing its expression [86] suggesting a link between AQP3 and oestrogen receptor positive breast cancer. The possibility to use AQP3 as a prognostic marker in breast cancer can be attributed to its role in cell migration, which is facilitated by channelling both, water and glycerol, further resulting in lamellipodia formation and consequently, cell movement and migration [86,87]. Studies confirmed that AQP3 overexpression increased cell migration and invasion [86] for oestrogen-receptor positive breast cancer cells, as well as for keratinocytes [87]. Further, in keratinocytes, AQP3 facilitates glycerol transport into the cell as well, resulting in ATP generation and proliferation [88]. The fact that AQP3 facilitates the transport of both, water and glycerol, together with H_2_O_2_ puts AQP3 high on the list of potential targets for tumour therapy. At the same time, signalling pathways and cellular processes affected by changes in AQP3 levels need to be clarified. Water and glycerol transport affect migration and metabolic processes (especially lipid metabolism), while H_2_O_2_ is the one that affects signalling pathways. H_2_O_2_ fuels several signalling pathways in the cell and a channel that can facilitate H_2_O_2_ transport is a potential candidate that provides some level of control over those pathways. In support of this assumption is the study by Hara-Chikuma et al. [89] showing in keratinocytes that TNFα stimulus is facilitated through NADPH oxidase isoform 2 (NOX2) production of H_2_O_2_. AQP3 then transports H_2_O_2_ resulting in regulation (inhibition) of protein phosphatase 2A and activation of nuclear factor kappa B (NF-κB). Even more, C-X-C motif chemokine ligand 12 (CXCL12) stimulates H_2_O_2_ transport across the membrane by AQP3 in breast cancer cells MDA-MB-231 and DU4475 [90]. The oxidation of phosphatase and tensin homolog/protein tyrosine phosphatase 1B (PTEN/PTP1B) occurs due to H_2_O_2_ followed by activation of the AKT pathway and again, resulting in cell migration. Knockdown of AQP3 impairs this process thereby supporting the role of AQP3 in migration [90]. The signalling pathways are summarized in Figure 3.

The need to study pathways affected by AQP3 overexpression and mechanisms of action is reflected by the finding that Auphen, aquaporin gold-containing inhibitor, blocked glycerol transport quite efficiently (about 90% inhibition), while water transport was blocked only partially (20% inhibition) [91]. Having in mind that AQP3 also facilitate H_2_O_2_ transport across the plasma membrane [92,93] and structural similarities between H_2_O_2_ and water [94], inhibitors should be carefully examined for their ability to block AQP3 all three molecules channelled via AQP3. For these reasons, the fact that an inhibitor can block glycerol, but not water transport implies that H_2_O_2_ transport can also be unaffected or affected at smaller rate suggests activation and modification of cellular processes in an undesired direction, driving to progression rather than to regression of the tumour. Interestingly, although it is well known that AQP3 channels H_2_O_2_, there are several papers on the effect of aquaporins in general on the antioxidative defence system, specifically NRF2 transcription factor, regardless of the disease [95,96,97]. In breast cancer cell lines, MCF7, SUM159 and SkBr3, AQP3 was the most expressed aquaporin, and in HER2 positive cells it was upregulated together with NRF2 by H_2_O_2_ [95] implying for then need to study effects of AQP3 overexpression in relation to the parts of antioxidative system.

## 6. AQP5 and Breast Cancer

AQP5 is involved in normal mammary development and milk production, as well as in breast carcinogenesis [80,98]. Jung et al. showed that silencing of AQP5 or induction of hyperosmotic stress to MCF-7 cells decreases the expression of AQP5 and negatively affects cell proliferation and migration. Additionally, AQP5 expression in benign tumours and invasive ductal carcinoma showed different patterns, expression of AQP5 in apical domains of ductal epithelial cells vs. increased expression in cancer cells with the loss in ducts accompanied with the loss of apical polarity, thus suggesting its role in breast cancer progression [99]. In triple-negative breast cancer (TNBC) patients, markedly higher expression of AQP5 and AQP3 was observed in cancer tissue than in adjacent normal tissue. Overexpression of AQP5 was mainly observed within Ki-67 high TNBC samples and, together with the higher expression of AQP3, associated with the more progressive disease with poorer overall survival proposing their co-expression as an independent prognostic marker in TNBC patients [84]. Additionally, overexpression of AQP5 was associated with worse outcomes in early breast cancer patients regardless of tumour type and stage, suggesting it as an independent prognostic marker of survival, particularly in hormone receptor-positive patients who underwent curative surgery [100]. 

**Figure 4:**
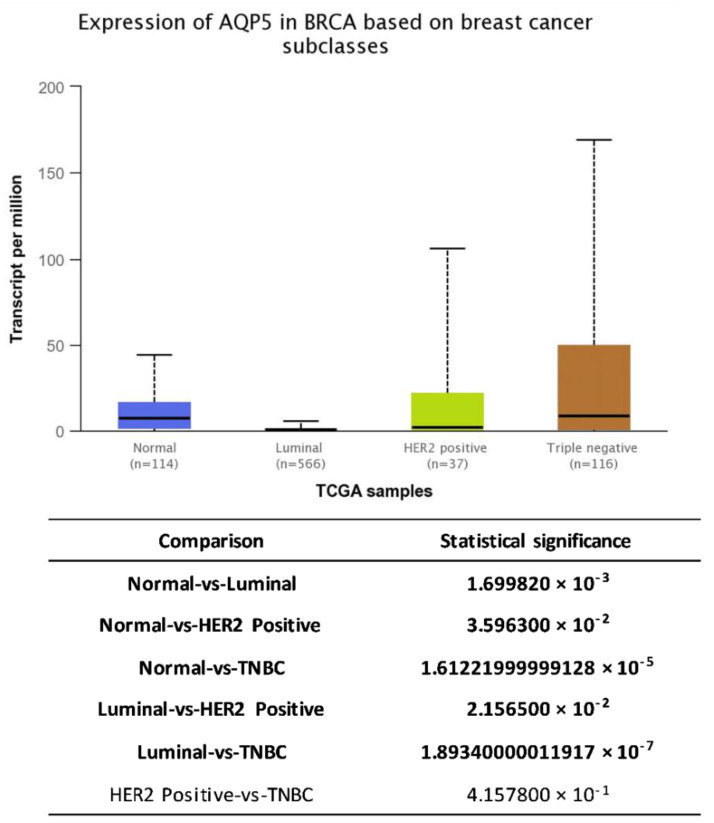
The expression of AQP5 in normal and breast cancer tissue subdivided according to expression of hormone and HER2 receptors retrieved from UALCAN [83].

Analysis of genomics data from The TCGA project, freely available from web-portal UALCAN [83], revealed significantly different gene expression pattern of AQP5 in normal (median 7.366 transcripts per million (TPM) (0–44.252 TPM)) vs. different subclasses of breast cancer (luminal–0.34 TPM (0–5.848 TPM), HER2-positive-1.79 TPM (0–105.814 TPM), TNBC-8.469 TPM (0–169.313 TPM); Figure 4).

Therefore, in our previous study, we examined how oxidative challenge (known contributor to breast carcinogenesis and a mechanism of effective anticancer therapy too [101]) affects lipid profile, levels of oxidative stress mediators and NRF2, the expression pattern of AQP1, AQP3, AQP5, and sensitivity to H_2_O_2_ in three breast cancer cell lines (representing hormone-positive (MCF-7), HER2-positive (SkBr-3) and TNBC (SUM 159)) [95]. Levels of polyunsaturated fatty acids (PUFA) were cell-type dependent with the highest observed in triple-negative SUM 159 cell line, and along with lower NRF2 levels may explain their higher sensitivity to H_2_O_2_. AQPs’ expression pattern was cell-type specific, also. While AQP3 was the most expressed isoform in all cell lines tested, exposure to H_2_O_2_ increased AQP3 expression in MCF-7 and SkBr-3 cells whereas in SUM 159 AQP3 was decreased. At the same time, the expression of AQP5 and AQP1 was similar in SUM 159 and SkBr-3, increased upon oxidative challenge, while decreased in MCF-7. 

Aside from being a water channel, a study by Rodrigues et al. has shown a highly efficient peroxiporin activity of AQP5, with external oxidative stress stimuli rescuing the suppression of cancer cells’ migratory ability induced by AQP5 silencing. Hence, the authors highlighted the role of AQP5 in dynamic fine-tuning of the intracellular levels of H_2_O_2_ [102] that are important for redox signalling and regulation of cell fate [103]. Thus AQP5 might show promise in anticancer therapy. Indeed, the discovery of three AQP5-regulating miRNAs (miR-1226–3p, miR-19a-3p, and miR-19b-3p) that, by decreasing the translation of AQP5, reduce breast cancer cell migration, supports its further investigation as a possible therapeutic target [104]. 

The connection of ROS and AQP5 was also observed in a study by Oh et al. They studied how hypercholesterolemia and the inhibition of xanthine oxidase (ROS-generating enzyme) affects breast cancer progression in both, in vitro and mouse xenograft model. Hyperlipidemic conditions were shown to contribute to ROS production, breast cancer progression, and MAPK activation, whereas treatment with febuxostat, xanthine oxidase inhibitor, by diminishing ROS levels and AQP5 expression, mitigated proliferative and migratory ability of breast cancer cells, as well as pulmonary metastases [105].

Whether the involvement of AQP5 in breast carcinogenesis is causative or merely a consequence of breast cancer cell’s need to grow, involving metabolic reprogramming and redox signalling including ROS, particularly H_2_O_2_, still needs to be elucidated. 

## 7. Conclusions

Aquaporin 3 and aquaporin 5 are upregulated in breast cancer and certainly support processes leading to breast cancer growth and metastasis. Current knowledge indicates that these two aquaporins as potential biomarkers of breast cancer malignancy making them potential therapeutic targets. In order to define AQP3 and AQP5 as targets for cancer treatment, it is needed to thoroughly study all possible aspects and pathways affected, as inadequate inhibition or stimulation of each AQP could drive cancer cells to the more malignant phenotype. One of the currently neglected aspects is certainly, crosstalk with the antioxidative system, especially, as AQP3 and AQP5 channel H_2_O_2_ which then plays an active role in signalling pathways.
